# To Dr. Batty

**Published:** 1803-06-01

**Authors:** J. Abernethy


### To Dr. BATTY.

DEAR SIR,

THERE is an omission in the description of the gorget, contained in the last number of the Journal, which I feel obliged to request you to supply. After speaking of the ill consequences of the cutting edge being of too small or of too great extent, it is added, (page 394, line 14) Three quarters of an inch is the distance to which I have directed that the cutting edge should extend, from the beak in the *transverse direction* in a gorget for operating on an adult subject. The words printed in italics, were interlined in my manuscript, and have not been inserted in the Journal. Perhaps this description is not sufficiently explicit; but I thought it would be intelligible to persons already acquainted with the general form and use of these instruments. It might have been said that the most projecting part of the cutting edge, should not extend further than three-quarters of an inch in the tranverse direction, from a line drawn length-wise along the middle of the instrument, from the beak to the handle. There was also a little diagram connected with the drawing that I sent you, which is omitted in the plate. It represented, by a dotted line, a portion of a circle drawn by putting one leg of a pair of compasses at the end of the cutting edge, next the beak, and opening them to the exact depth of the groove of the staff, and by describing with the other the circle, to a segment of which the beak should be ground so as exactly to correspond. In the plate, the most projecting part of the beak is not drawn to represent an exact segment segment of a circle. It is difficult to describe an instrument so precisely and fully, as to leave no room for misconception, I have therefore preferred a general and concise description of the gorget, to one more minute and laboured, and which, after all, might still be liable to be misunderstood. I am, &c.

*May* 13, 1803. J. ABERNETHY.

